# Therapeutics to tackle Omicron outbreak

**DOI:** 10.2217/imt-2022-0064

**Published:** 2022-06-09

**Authors:** Vivek P Chavda, Eswara Naga Hanuma Kumar Ghali, Murali M Yallapu, Vasso Apostolopoulos

**Affiliations:** ^1^Department of Pharmaceutics & Pharmaceutical Technology, LM College of Pharmacy, Ahmedabad, Gujarat, 380008, India; ^2^Department of Immunology & Microbiology, School of Medicine, University of Texas Rio Grande Valley, McAllen, TX 78504, USA; ^3^South Texas Center of Excellence in Cancer Research, School of Medicine, University of Texas Rio Grande Valley, McAllen, TX 78504, USA; ^4^Institute for Health & Sport, Victoria University, Melbourne, Victoria, 3030, Australia; ^5^Immunology Program, Australian Institute for Musculoskeletal Science (AIMSS), Melbourne, Victoria, 3021, Australia

**Keywords:** convalescent plasma, COVID-19, monoclonal antibody, mutation, Omicron, SARS-CoV-2

## Abstract

In this commentary, the authors have focused on the mutational impact of the Omicron variant on the current therapeutics to manage #COVID19.

## Graphical abstract

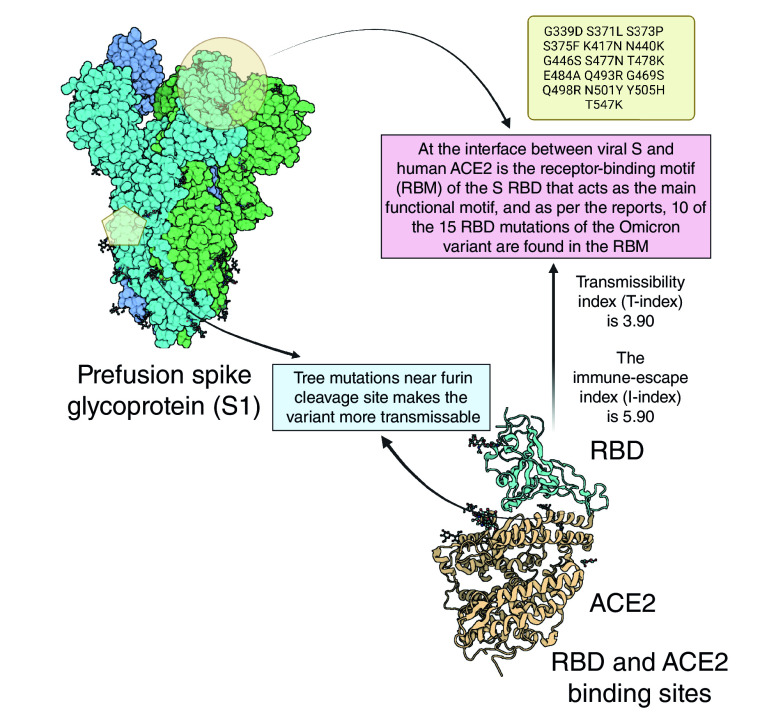

Spike protein mutational impact in Omicron variant.

Emerging variants of the SARS coronavirus 2 (SARS-CoV-2) are creating significant worry due to their potential to evade innate or vaccine-induced protection as well as current therapies. It is vital to ascertain the influence of these polymorphisms on pandemic prevention, particularly in medicines and vaccines [1]. RNA viruses, particularly coronaviruses, rapidly acquire mutations when compared with DNA viruses [2]. One of the earliest SARS-CoV-2 mutations described was an aspartic acid-to-glycine substitution at amino acid position 614 of the spike (S)-protein (referred to as D614G) [3]. New variants may pose issues for disease management due to increased disease transmissibility and virulence, as well as an increased ability to cause disease in people who received immunizations that contained the variant [4].

Now that the world has already experienced the devastating impact of Delta and Delta plus variants of SARS-CoV-2, the disease is spreading its wings through a new variant of concern – Omicron [5]. Multiple mutations in the recently emerged SARS-CoV-2 variants, predominantly in their surface S-glycoproteins, lead to the enhancement in transmissibility and resistance to neutralizing antibodies [6]. Omicron variant has more than 52 noted mutations, with 30 mutations identified in the viral S-protein only [7]. It is clearly understood, from the currently published research findings, that this emerging variant demands new vaccines be designed, as the currently approved vaccines under emergency use authorization (EUA) are not providing total immune protection [6,8–13]. Hence, in this tough time, a proper therapeutic strategy needs to be established for the Omicron variant. Numerous studies have provided the present view of how SARS-CoV-2 S-protein mutations impact neutralization [14,15]. S mutations also pose a risk to the efficacy of current COVID-19 vaccines and antibody resistance [16]. According to WHO, there are several lineages of Omicron; of them, BA.1, BA.1.1 (or Nextstrain clade 21K) and BA.2 (or Nextstrain clade 21L) are the most common. However, studies are still going on to understand the advantage of BA.2 over BA.1 in terms of growth, transmissibility and other related antigenic strategies. Studies by Bhattacharya *et al.* [17] stated that the highest number of notable mutations are found in S-glycoprotein. The important properties such as higher infectivity, transmutability, higher binding affinity, antibody resistance and different mutations in S-protein from various published data are tabulated in Table 1. A total of four sub-lineages of Omicron are identified, namely BA.1, BA.1.1, BA.2 and BA.3 [18].

**Table 1.** Mutations, their positions and properties of spike-protein in Omicron variant.

Spike-protein region	Code of mutation	Amino acid mutation	Outcome
RBD region	G339D	Gly^339^→Asp	Enhanced ACE2 binding of virus
	S371L	Ser^371^→Leu	Antibody evasion
	S373P	Ser^373^→Pro	Making virus more infectious
	S375F	Ser^375^→Phe	Making virus more infectious
	K417N	Lys^417^→Asn	Making virus more infectious
	N440K	Asn^440^→Lys	Antibody evasion and increased infection
	G446S	Gly^446^→Ser	Antibody evasion and increased infection
	S477N	Ser^477^→Asn	Decreases protein stability and increases infection vulnerability
	T478K	Thr^478^→Lys	Decreases protein stability and increases infection vulnerability
	E484A	Glu^484^→Ala	Decreases protein stability and increases infection vulnerability
	Q493K	Gln^493^→Lys	Higher antibody resistance
	G496S	Gly^496^→Ser	Decreases protein stability and increases infection risks
	Q498R	Gln^498^→Arg	Decreases protein stability and increases infection risks
	N501Y	Asn^501^→Tyr	Enhanced ACE2 binding of virus and immune escape
	Y505H	Tyr^505^→His	Decreases protein stability and increases infection risks
Other than RBD region	A67V	Ala^67^→Val	–
	Δ69–70	Deletion mutations	–
	T95I	Thr^95^→Ile	–
	G142D	Gly^142^→Asp	–
	Δ143–145	Deletion mutations	–
	Δ211	Deletion mutations	–
	L212I	Leu^212^→Ile	–
	ins214EPE	Insertions of Glu, Pro and Glu amino acids	–
	T547K	Thr^547^→Lys	Stabilizes RBD in down confirmation
	D614G	Asp^614^→Gly	Increased the viral fitness and higher transmissibility
	H655Y	His^655^→Tyr	Increases viral transmissibility
	N679K	Asn^679^→Lys	Increases viral transmissibility
	P681H	Pro^681^→His	Increases viral transmissibility
	N764K	Asn^764^→Lys	–
	D796Y	Asp^796^→Tyr	–
	N856K	Asn^856^→Lys	–
	Q954H	Gln^954^→His	–
	N969K	Asn^969^→Lys	–
	L981F	Leu^981^→Phe	–

ACE2: Angiotensin-converting enzyme 2; RBD: Receptor-binding domain.

Understanding the continuous evolution of SARS-CoV-2 is crucial to controlling the pandemic. There are substitutions – such as G339D, S371L, S373P, S375F, K417N, N440K, G446S, S477N, T478K, E484A, Q493R, G496S, Q498R, N501Y and Y505H – identified in the receptor-binding domain (RBD). They contribute to an advantage to Omicron over Delta in terms of higher human angiotensin-converting enzyme 2 (ACE2) affinity and interfacial stability at RBD. A study of binding interactions, conducted by Lupala *et al.* [19] using atomistic molecular dynamics simulations, revealed that binding of Omicron S-protein is stronger to the human ACE2 protein than the original strain. This might be attributed to changes at the ACE2–RBD junction that improved hydrophobic interactions and suppressed solvent-specific surface area [20,21]. Therefore, many of the approved monoclonal antibodies (mAbs) became ineffective or showed less activity against the Omicron variant. In addition, antigen, molecular, and serology tests are also affected by heavy mutations due to the differences in the inherent design of each test. The viral replication of the Omicron variant is through an endocytic pathway rather than the transmembrane serine protease 2 pathway as that of other variants of concern [22].

Conventional escape mutation research contains mutations that originate in virus populations that are exposed to either mAbs or convalescent plasma comprising polyclonal antibodies, targeted characteristics of specific mutations, and broader research of either huge numbers of propagating variants or all feasible amino acid replacements in the RBD [23]. The difference is that N439K confers immunological escape via enhanced ACE2 affinity instead of adversely challenging antibody epitope recognition that may be less sensitive to discovering immune evasion mutations of this type [24]. According to a researcher at Hong Kong University [25], it is important to note that the severity of disease in humans is not determined only by virus replication but also by the host's immune response to the infection, which may lead to dysregulation of the innate immune system, that is, a cytokine storm. Mannar and colleagues [26] performed cryo-electron microscopy based structural analysis of Omicron and concluded thet there is an increase in antibody evasion, together with retention of strong interactions at the ACE2 interface, thus represent important molecular features that likely contribute to the rapid spread of the Omicron variant. Zhang *et al.* investigated 28 blood samples from COVID-19 convalescent patients and discovered an 8.4-fold reduction in neutralization against the Omicron strain when matched to the D614G-susceptible strain [27]. According to Wang *et al*., Omicron demonstrates an exceptional immunological escape from past infections and immunization with two doses of the vaccine CoronaVac when compared with prior spontaneously evolving SARS-CoV-2 variants Alpha, Beta, Gamma, Delta, Lambda and Mu. As per their findings, the Omicron variant was 1.4-times more resistant to neutralization by convalescent plasma than the Mu variant. Furthermore, the Omicron was two-times more resistant to neutralization by convalescent plasma than the Beta variant [28]. A multicentre, double-blind, phase III trial (NCT04545060) was conducted by Vir Biotechnology and GlaxoSmithKline to evaluate the efficacy of sotrovimab (500 mg) against Omicron on 868 non-hospitalized patients.

Globally, the Omicron variant is circulating, rapidly becoming dominant and is considered an important reason for the antibody evasion activity in the convalescent plasma or vaccinated individuals [29]. A study conducted on the Omicron pseudovirus in the BHK21-ACE2 cell line and Vero-E6 cells found that this strain is more virulent than the Delta strains. It also displays an eight- to ten-fold reduction in neutralization in the neutralization assay using serum samples from Wuhan convalescents that were taken 1 year after infection. In addition, after three vaccination doses, the convalescents' protection against Omicron variants improved. This emphasizes the need for booster doses in combating the threat of SARS-CoV-2 immunological divergence [30]. A study by Gruell *et al.* [31], in longitudinal cohorts of convalescent and vaccinated individuals, demonstrated that booster immunizations critically improved the humoral immunity against the Omicron variant. Further investigation on 28 blood samples from COVID-19-recovered individuals infected with the primary virus strain versus pseudotyped Omicron found an 8.4-fold reduction in the median effective dosage to 66 when matched to the D614G reference strain (median effective dosage = 556) [27]. A comparative study on the binding capability of the RBD and antibodies in the sera of COVID-recovered patient and sera of the vaccinated individual after two doses of the inactivated vaccine (Make: Sinopharm WIBP) revealed that the Omicron variant may evade antibodies as well as cause a substantial decrease in the binding potential of its RBD, especially in comparison to the Delta variant [32]. The Omicron variant was neutralized to a much lesser extent in the serum samples collected from vaccinated persons compared with Alpha, Beta or Delta variants, although prominent cross-neutralization was noticed against other variants [8]. An investigation on the neutralization of the virus and S-protein binding efficacy of sera from convalescent double-vaccinated, convalescent, convalescent boosted individuals against wild-type, double mRNA-vaccinated, Omicron SARS-CoV-2 isolates, mRNA-boosted and Beta (B.1.351) recovered patients showed that there was an undetectable or very low neutralizing activity of sera from double-vaccinated and convalescent participants [33].

mAbs are the immune molecules produced in the laboratory that can enhance the immune system's attack on cells. They are now recommended and available in some countries to treat patients at high risk of COVID-19. Treatment with mAb can limit the amount of SARS-CoV-2 within the body and block its entry and attachment to the human cells. However, heavily mutated RBD (15 mutations) of the SARS-CoV-2 S-protein of the Omicron variant potently evades approved COVID-19 mAbs and exhibits high affinity toward binding to the ACE2 receptor [34]. Only 3 out of 29 mAbs hold unaltered potency in *in vitro* neutralizing activity against Omicron, including the S2K146 antibody [35]. A fraction of broadly neutralizing sarbecovirus mAbs, such as sotrovimab, S2X259 and S2H97, are capable of neutralizing Omicron by recognizing antigenic sites located outside the receptor-binding motif [36]. A previous study on 247 human anti-RBD neutralizing antibodies demonstrated that when neutralizing antibodies target the sarbecovirus conserved region, they will remain most effective [37]. In another research work, Hoffman reported that the Omicron variant is resistant to most therapeutic antibodies but remains susceptible to inhibition by sotrovimab [38].

The FDA revised the dosage of Evusheld™ (tixagevimab co-packaged with cilgavimab) to 300 mg of cilgavimab and 300 mg of tixagevimab as an EUA against Omicron subvariants BA.1 and BA1.1. In addition, the FDA issued an EUA for bebtelovimab for the treatment of mild to moderate COVID-19 patients. However, it was not authorized for hospitalized patients due to the worse clinical outcomes. Clinically used anti-RBD mAbs such as VIR-7831 (sotrovimab), COV2-2196 and COV2-2130 (AstraZeneca), REGN10933 and REGN10987 (Regeneron), LY-CoV555 and LY-CoV016 (Eli Lilly), and CT-P59 (Celltrion) in Vero-TMPRSS2 and Vero-hACE2-TMPRSS2 cells revealed that REGN10987, REGN10933, LY-CoV016, LY-CoV555 and CT-P59 completely lost neutralizing activity against the B.1.1.529 virus. However, the effect was reduced by 12-fold with COV2-2130 and COV2-2196 combination and minimally affected in the presence of S309 [39]. Remdesivir, molnupiravir and nirmatrelvir were found to be 50% effective against the Omicron variant in mild to moderate infections [40]. Together, these investigations suggest that the Omicron variant indeed speeds up the shift in the treatment regimen for COVID-19. These developments would make COVID-19 more manageable than in the previous 2 years of the pandemic. Research is needed for the safe usage of anti-SARS-CoV-2 mAbs in pregnant and lactating women, as most of the IgG crosses the placental barrier. Similarly, there is no evidence of the clinical benefits of such mAb treatments in B-cell immunodeficiency or other immunodeficiencies [41]. The RECOVERY study, BLAZE study and ACTIVE-3 studies' results mainly suggest mAb usage in non-hospitalized mild to moderate symptomatic COVID-19 patients. However, more data and research is needed to prove its clinical benefits in severe COVID-19 patients [42–44].

Due to the high mutation rate of the S-protein of SARS-CoV-2 in the emerging variants, the neutralizing antibodies present in the individual previously infected with other variants of SARS-CoV-2 or vaccine-induced neutralizing antibodies are not able to provide sufficient immune protection. SARS-CoV-2 specific mAbs are generally designed with S-protein as a target, and that ultimately compromises the efficacy of mAbs against emerging new variants, especially in severe cases. The ultimate conclusion from all the above research findings is the designing of variant-specific therapy and vaccines for better immune protection. To seize the spread of variant, the intranasal variant-specific vaccine is a better option as it offers localized IgA-mediated immune protection [45,46]. Intranasal delivery of therapeutic mAbs based on nano-drug delivery is also a niche sector for researchers, especially for infectious communicable diseases.
